# The Woman Who Lives without Eating

**Published:** 1859-04

**Authors:** L. E. Whiting

**Affiliations:** Saratoga Springs


[From the Boston Medical and Surgical Journal.]

### "*The Woman who lives Without Eating.*"

Messrs. Editors,—Having seen several notices of the abov case in newspapers, and hearing still more from persons who had seen this "living miracle," I made her a visit a few days since, for the purpose of learning more fully and accurately her real state.

Her name is Betsy Hays. She lives in the town of Horicon, Warren County—some sixty miles from our village (Saratoga Springs). I found her lying upon her back, with her head drawn so far over that I could only see her chin, her face looking back toward the walls of the room. The first impression on seeing her, was such as one gets from a severe case of hysteria. She seemed generally convulsed, tremulous, and rigid. She had been in this condition for a long time.—I learned from those who often see her, as well as from her husband, that she usually presents the same appearance. She looks fresh, and is not emaciated. Her body is warm, and the skin very clear and soft. Her respiration is very irregular. The pulse was small and threadlike, but I found it difficult to ascertain accurately its frequency, so constantly were the muscles moving. I should think it something over a hundred. Her husband (who seems a simple, honest-minded man) told me her age was 28, and that she had never been sick—until about four years ago, when her present illness commenced—except at hear confinements. She is the mother of four children.

Four years ago, she was taken with pain in her back and hips, inability to walk, in fact with all the symptoms of falling of the womb. This was in November. In June following she became worse, lost her eyesight, and in July was taken with spasms. These increased in violence. They would sometimes last for weeks; then she would recover consciousness, converse, take some light food, and again relapse into the same state.

For the space of two years from last June, she has not taken any food of any kind or description. She took a tablespoonful of cold water once in a few days, until February, *two years* ago—since which time she has *neither ate nor drank.* Her respiration, which I said was irregulur, is at times apparently suspended for and hour and even more than that, often for fifteen or twenty minutes. Her face and neck become very livid at such times, and there is a choking sound in the throat. During the spasms, she sometims raises herself up in bed, her head still thrown backward, and then down again on the pillow with great force.

She has had no dejections from the bowels, nor any secretion of urine for the *last two years.* Her feet are cramped, and most of the time the left foot rest upon the instep of the right. Her toes are drawn under and imbedded in the flesh, and her feet are drawn under to such a degree that they present almost the appearance of *club feet.* The left hand, with the fingers tensely flexed, is pressed against the left side, where it permanently remains. It required great strength to raise it an inch from her body. The right hand is cramped and the fingers flexed, but she is continually striking her stomach with it, when the spasms are violent. At such times her jaw is dislocated and thrown into its place with great rapidity, making a noise that could be heard across the room.

Such is the state of this extraordinary woman. We can dispose of the case very easily by saying that it is one of "successful fraud and deception," as the State Medical Society have just done at their annual meeting in Albany.

The Society, no doubt, came to such conclusions by the evidence placed before them; but as one of your best jury lawyers in Massachusetts said, "he wanted to *see* the testimony as well as hear it," so, in this case, one cannot tell all about it. You want to see it; you want to see the room, the husband and children, and talk with them, the neighbors, and those who have seen the most of this wonderful woman; you want to put your hands on those rigid muscles, and then watch the suspended breath the whole body quivering with the tenseness of the convulsions until you are obliged to turn away for your own relief. I say one wants to see all these phenomena, then learn that there can be no motive for the deception, no reward for all this suffering, no object, no inducement. One wants to see all this, and he will be strongly inclined to call it *no fraud.*

An article in *Blackwood's Magazine*, and republished in the April number of the *Eclectic Magazine*, on the "Phenomena of Huner and Thirst," relates several such cases, some of which lived from four to eight years. They were mostly reported in the *Philosophical Transactions.* The writer disposes of them in this manner: "It is rather startling to find so learned a physiologist as M. Bérard recording such cases, and trying to explain them. The possibility of deception and exaggeration is so great, that we are tempted to reject almost every one of these cases, rather than reject all physiological teachings." But M. Bérard says: "Admitting that there has been deception in some of these cases, and that the love of the marvellous has presided over the narration of others, we cannot refuse to believe that some are authentic. Every year such cases are registered."

L. E. WHITING.

*Saratoga Springs, Feb.* 22, 1859.

